# Can the Results of Biodiversity-Ecosystem Productivity Studies Be Translated to Bioenergy Production?

**DOI:** 10.1371/journal.pone.0135253

**Published:** 2015-09-11

**Authors:** Timothy L. Dickson, Katherine L. Gross

**Affiliations:** 1 Department of Biology, University of Nebraska-Omaha, Omaha, Nebraska, United States of America; 2 W.K. Kellogg Biological Station and Plant Biology Department, Michigan State University, Hickory Corners, Michigan, United States of America; Helmholtz Centre for Environmental Research - UFZ, GERMANY

## Abstract

Biodiversity experiments show that increases in plant diversity can lead to greater biomass production, and some researchers suggest that high diversity plantings should be used for bioenergy production. However, many methods used in past biodiversity experiments are impractical for bioenergy plantings. For example, biodiversity experiments often use intensive management such as hand weeding to maintain low diversity plantings and exclude unplanted species, but this would not be done for bioenergy plantings. Also, biodiversity experiments generally use high seeding densities that would be too expensive for bioenergy plantings. Here we report the effects of biodiversity on biomass production from two studies of more realistic bioenergy crop plantings in southern Michigan, USA. One study involved comparing production between switchgrass (*Panicum virgatum*) monocultures and species-rich prairie plantings on private farm fields that were managed similarly to bioenergy plantings. The other study was an experiment where switchgrass was planted in monoculture and in combination with increasingly species-rich native prairie mixtures. Overall, we found that bioenergy plantings with higher species richness did not produce more biomass than switchgrass monocultures. The lack of a positive relationship between planted species richness and production in our studies may be due to several factors. Non-planted species (weeds) were not removed from our studies and these non-planted species may have competed with planted species and also prevented realized species richness from equaling planted species richness. Also, we found that low seeding density of individual species limited the biomass production of these individual species. Production in future bioenergy plantings with high species richness may be increased by using a high density of inexpensive seed from switchgrass and other highly productive species, and future efforts to translate the results of biodiversity experiments to bioenergy plantings should consider the role of seeding density.

## Introduction

Biofuel production has increased rapidly in the USA [1], partially due to the conversion of grasslands to row-crop agriculture [2] and the resulting increases in corn acreage [3]. While grain crops can produce high biomass, they provide few other ecosystem services [4], and the conversion of grasslands to row-crop agriculture has detrimental environmental consequences such as increased greenhouse gas emissions, increased fertilizer usage, and reduced habitat availability [5–7]. As an alternative to annual crops such as corn and soybeans, perennial bioenergy crops have the potential to provide high bioenergy production [8–11] and other ecosystem services [4,12,13].

A number of experimental studies in grasslands show that high diversity plantings produce more biomass than monocultures [14]. This has led some researchers to propose that bioenergy plantings using high species diversity should increase production and provide carbon-negative biofuels with minimal fertilizer and pesticide inputs [12,15]. However, past experimental studies have been designed to examine the theoretical relationship between species richness and production and have not included the practical issues that farmers will face in planting and maintaining crops for bioenergy production. We have identified some characteristics of biodiversity-productivity studies that limit their ability to inform bioenergy plantings. To clarify our terminology, most biodiversity-productivity studies focus on “richness” (number of species), but a few focus on “biodiversity” or “diversity” (measure combining number and relative abundances of species). We therefore use the terms “biodiversity” or “diversity” when discussing past biodiversity-productivity studies.

Experimental biodiversity studies prevent invasion of non-planted species by hand-weeding, whereas agricultural bioenergy plantings would allow low abundances of weeds or use herbicides or tillage to control weeds. The absence of hand-weeding will reduce differences between species richness in monoculture and high diversity plantings because non-planted species can (and do) invade these systems. Roscher et al. [16] show that planted species decline in abundance through time in the presence of weeds. Pfisterer et al. [17] show that after weeding is ceased in a biodiversity experiment, plant invasion can cause the diversity gradient to disappear over time and can remove differences in biomass production between treatments.

In addition, bioenergy plantings are likely to only use highly productive species in monoculture whereas, for experimental rigor, biodiversity studies have used both productive and unproductive species in monocultures and mixtures. Thus, some of the increase in biomass production between monocultures and more diverse mixtures in biodiversity experiments have been attributed to the sampling effect, in which more diverse mixtures are more likely to include highly productive species and, consequently, higher community-level biomass yields [18]. However, in bioenergy plantings the sampling effect will play almost no role in biomass production because all monocultures and mixtures will contain highly productive species. Even in biodiversity experiments, diverse mixtures often do not out-produce highly productive monocultures. A meta-analysis of 83 biodiversity experiments found that the most diverse mixture does not significantly outperform the most productive monoculture in 88% of experiments [19]. Also, an observational study by Adler et al. [20] shows that highly productive planted grasslands tend to contain fewer species than less productive grasslands, suggesting that the most productive sites may contain few species in reconstructed grasslands (even though Henschell et al. [21] find the opposite result). Other studies have shown that increased biodiversity can have other benefits such as increased stability in production and reduced invasion of new species [22].

There is a critical need to determine whether the results of past biodiversity studies can be translated to bioenergy plantings because even after considering the issues outlined above, diverse plantings may still produce more biomass and ecosystem services than monocultures, assuming processes such as complementary resource use operate [4,12,23]. We utilized data from established fields used for conservation plantings (Conservation Reserve Program plantings or similar) and a bioenergy crop experiment to evaluate the potential for systems with greater species richness to produce more biomass than monocultures under more realistic agricultural management. In both the field and experimental plantings, monocultures were planted with switchgrass (*Panicum virgatum*), a native C_4_ grass that has high potential as a bioenergy crop because of its high productivity and broad geographic range in North America [24]. The treatments with greater species richness were planted with mixtures of prairie forbs and grasses that are native to the Upper Midwestern USA. None of the plantings were weeded after planting and the methods used to manage the sites were similar to those used in bioenergy plantings (see below). Sampling of the established fields and experimental plots was done as part of the sustainability research thrust of the Great Lakes Bioenergy Research Center (GLBRC) and the Long Term Ecological Research (LTER) agricultural systems at the W.K. Kellogg Biological Station (KBS). We predicted that biomass production would increase with planted richness, though to less of an extent than past biodiversity-productivity studies.

## Materials and Methods

For the GLBRC field surveys (private land), the owners of the land gave permission to conduct the study on these sites. For permission to sample from the LTER experiment, we received Site Use Request #213 from the Kellogg Biological Station.

### GLBRC Field Surveys

We surveyed species composition and above-ground biomass production from 10 southern Michigan fields planted to switchgrass (*Panicum virgatum*) monoculture and 10 fields planted to moderately diverse prairie [4,25]. We hereafter refer to these surveys as “GLBRC field surveys”. The survey sites had been established an average of 10 years (switchgrass) or 8 years (prairie) at the time of sampling and varied in size from 1.0 to 12.4 ha. The plantings were located across southern Michigan along a 320 km transect from Lake Michigan to Lake Huron and were created by farmers and conservation groups [25] for erosion control or wildlife habitat. Plantings were either part of the Conservation Reserve Program (CRP) or were planted similarly to CRP sites (details of the site characteristics can be found in Table 1 and S1 Table). Historical photos shown in Google Earth (Google Inc., Mountain View, CA, USA) from the 1990’s show that many sites were in row-crop agriculture before planting, and those sites that had already been planted to switchgrass or prairie in the earliest photos would have been agricultural sites before planting (i.e. none of the sites were remnant prairies). Native grass CRP sites are generally planted to switchgrass [26] or to mixtures containing three or more grass species and two or more forb species [27].

**Table 1:** Site characteristics for the GLBRC field surveys. Details of each site and the average biomass collected from hand harvests in the two years of sampling; 1 Mg ha^-1^ = 892.2 pounds acre^-1^.

Year	Habitat	Nearest town	Location	Hectares	Monthly temperature range (°C)	AVG Precip (cm)	Average biomass (Mg per hectare)
2008	switchgrass	Cassopolis, MI	41.915 N; 85.978 W	4.13	-9 to 28	103.7	13.99
2008	switchgrass	Kalamazoo, MI	42.264 N; 85.647 W	2.07	-8 to 29	95.2	9.81
2008	switchgrass	Middleville, MI	42.644 N; 85.493 W	2.10	-9 to 28	95.3	6.25
2008	prairie	Marcellus, MI	41.954 N; 85.85 W	6.89	-9 to 29	98.1	11.14
2008	prairie	Oshtemo, MI	42.33 N; 85.671 W	6.36	-8 to 29	95.2	8.67
2008	prairie	Hastings, MI	42.536 N; 85.305 W	2.68	-9 to 28	95.3	9.25
2009	switchgrass	Fennville, MI	42.538 N; 86.111 W	7.24	-9 to 28	104.7	4.28
2009	switchgrass	Cassopolis, MI	41.915 N; 85.978 W	4.13	-9 to 28	103.7	8.15
2009	switchgrass	Kalamazoo, MI	42.264 N; 85.647 W	2.07	-8 to 29	95.2	4.67
2009	switchgrass	Constantine, MI	41.852 N; 85.608 W	3.58	-9 to 29	98.1	5.06
2009	switchgrass	Middleville, MI	42.644 N; 85.493 W	2.10	-9 to 28	95.3	5.12
2009	switchgrass	Grass Lake, MI	42.245 N; 84.229 W	4.14	-9 to 28	80.2	6.78
2009	switchgrass	Saginaw, MI	43.343 N; 84.109 W	3.34	-8 to 29	80.3	4.45
2009	switchgrass	Clifford, MI	43.292 N; 83.157 W	2.74	-10 to 28	84.9	7.59
2009	switchgrass	Bad Axe, MI	43.801 N; 82.891 W	4.68	-9 to 27	83.9	5.92
2009	switchgrass	Bad Axe, MI	43.77 N; 82.858 W	3.51	-9 to 27	83.9	5.46
2009	prairie	Fennville, MI	42.565 N; 86.096 W	12.43	-9 to 28	104.7	8.16
2009	prairie	Marcellus, MI	41.954 N; 85.85 W	6.89	-9 to 29	98.1	9.39
2009	prairie	Oshtemo, MI	42.33 N; 85.671 W	6.36	-8 to 29	95.2	6.95
2009	prairie	White Pigeon, MI	41.812 N; 85.583 W	1.01	-9 to 29	98.1	3.23
2009	prairie	Hastings, MI	42.536 N; 85.305 W	2.68	-9 to 28	95.3	7.69
2009	prairie	Charlotte, MI	42.652 N; 84.916 W	4.17	-9 to 28	85.9	8.53
2009	prairie	East Lansing, MI	42.801 N; 84.393 W	3.82	-8 to 28	81.8	6.99
2009	prairie	Vassar, MI	43.447 N; 83.543 W	3.63	-10 to 28	84.9	4.75
2009	prairie	Lapeer, MI	43.103 N; 83.237 W	3.50	-9 to 27	82.1	4.71
2009	prairie	Gagetown, MI	43.693 N; 83.208 W	6.35	-10 to 28	84.9	4.94

All sites were established from seed, although seeding densities at the time of planting are not known. We do not have information on fertilizer / pesticide inputs prior to planting, but none of the sites were fertilized after being planted to switchgrass or prairie. One of the switchgrass and three of the prairie sites had been burned in early spring 2007 or 2008, and two other prairie sites had been mown in 2007 (biomass not removed) [4]. All other sites had not been disturbed within two years of sampling. Analyses in Werling et al. [4] show that disturbance did not significantly affect plant biomass or species richness. Plant biomass and species composition sampling was done in two years (2008–2009) with 3 switchgrass and 3 prairie sites sampled in 2008 and 10 switchgrass and 10 prairie sites sampled in 2009. The sites sampled in 2008 were a subset of those sampled in 2009. At least one switchgrass and one prairie site sampled in 2009 and at least one prairie site sampled in 2008 was planted with wild-type Michigan seed. It is likely that at least some of the other sites were planted with cultivar seed typical of CRP (we do not have data on the varieties of seeds used in most plantings). Above-ground biomass was cut at the soil surface using hand clippers. Four 0.5m x 2m quadrats were harvested from each site, with each quadrat randomly located within each site. Harvests occurred in late August or early September, corresponding to peak biomass. The harvested material was sorted to big bluestem (*Andropogon gerardii*), Indiangrass (*Sorghastrum nutans*), switchgrass (*Panicum virgatum*), other grasses, or forbs. The sorted biomass was dried for 72 hours at 65°C and then weighed. On the same days as biomass harvests, visual surveys were conducted in 1 m^2^ circles located 2 m south of each quadrat (four surveys per site) to determine the number of species present, but not their relative abundances or evenness.

### LTER Experiment

To directly examine the relationship between species richness and productivity, we analyzed data from a subset of treatments in the KBS LTER Cellulosic Biofuels Diversity Experiment (further details can be found in S1 Table). We hereafter refer to this study as the “LTER experiment”. This experiment was established in spring 2008 by planting seeds of 1 to 30 native species into plots (9.1m x 27m) that had been sprayed with Roundup OriginalMax (glyphosate) applied at 2.3 L ha^-1^ with Ammonium Sulfate at 0.02 kg L^-1^ (3.82 kg ha^-1^) and had been tilled and disked in the fall and spring prior to planting. Prior to this planting the site had been used for experimental cropping systems that involved a variety of rotations and inputs. The plots were not weeded, but they were mowed in summer 2008 to reduce abundance of non-planted species.

The treatments were established to create a nested range of species richness. More species rich plantings contained the species present in less diverse plantings, plus additional species. Specifically, monocultures contained switchgrass, 2 species mixtures added Canada wild rye, 6 species mixtures added big bluestem, Indiangrass, Junegrass, and little bluestem, 10 species mixtures added 4 forb species, 18 species mixtures added 8 forb species, and 30 species mixtures added 12 forb species (Table 2 contains Latin names, identities, and seeding densities of all species). Each diversity treatment was replicated once in each of four blocks. In establishing these treatments, a similar total seed weight was used in all treatments (between 7.15 and 8.08 kg ha^-1^), except the switchgrass monocultures where 16.15 kg ha^-1^ of seed was added. Switchgrass was planted at lower density in the mixtures because of concerns that it would become dominant if planted at the rate used in monoculture (Table 2 and S2 Table).

**Table 2:** Seeding densities for LTER experiment. The seeding densities in kg ha^-1^ for every species planted into the LTER experiment. The columns show the richness levels of different treatments and the respective seeding densities.

common name	Latin name	1 sp.	2 sp.	6 sp.	10 sp.	18 sp.	30 sp.
switchgrass	*Panicum virgatum*	16.15	4.62	1.44	0.58	0.58	0.58
Canada wild rye	*Elymus canadensis*		3.46	1.62	1.15	0.87	0.58
big bluestem	*Andropogon gerardii*			1.15	1.15	0.87	0.58
Junegrass	*Koeleria cristata*			0.58	0.58	0.58	0.43
little bluestem	*Schizachyrium scoparium*			2.02	1.44	0.87	0.87
Indiangrass	*Sorghastrum nutans*			1.10	1.15	0.87	0.58
showy tick trefoil	*Desmodium canadense*				0.37	0.26	0.14
wild bergamot	*Monarda fistulosa*				0.37	0.26	0.14
stiff goldenrod	*Oligoneuron rigidum*				0.37	0.26	0.14
black-eyed Susan	*Rudbeckia hirta*				0.37	0.26	0.23
Canada anemone	*Anemone canadensis*					0.26	0.14
butterfly weed	*Asclepias tuberosa*					0.26	0.14
white wild indigo	*Baptisia alba*					0.26	0.14
round-headed bushclover	*Lespedeza capitata*					0.26	0.14
yellow coneflower	*Ratibida pinnata*					0.26	0.14
cup plant	*Silphium perfoliatum*					0.26	0.14
showy goldenrod	*Solidago speciosa*					0.26	0.14
New England aster	*Symphyotrichum novae-angliae*					0.26	0.14
leadplant	*Amorpha canescens*						0.14
common milkweed	*Asclepias syriaca*						0.14
tall coreopsis	*Coreopsis tripteris*						0.14
purple prairie clover	*Dalea purpurea*						0.14
Illinois tick trefoil	*Desmodium illinoense*						0.14
flowering spurge	*Euphorbia corollata*						0.14
common evening primrose	*Oenothera biennis*						0.14
gray goldenrod	*Solidago nemoralis*						0.14
smooth blue aster	*Symphyotrichum laeve*						0.14
hairy aster	*Symphyotrichum pilosum*						0.14
Ohio spiderwort	*Tradescantia ohiensis*						0.14
golden Alexanders	*Zizia aurea*						0.14

We used data on biomass production from 2010–2013 (3–6 years after planting) to examine the relationship between planted species richness and biomass production in unfertilized plots. In all four years, biomass production was determined from harvesting with a tractor-mounted mower in November, baling the biomass, and weighing the bales. We also determined biomass production from a September 2012 hand harvest of three treatments (monoculture, 6, and 18 planted species; from 0.5m x 2m quadrats). In both harvest methods, plants were cut 10 cm above the soil surface, comparable to the height at which the tractor-mounted harvester removed biomass. For tractor-harvested biomass, moisture content was measured and used to calculate the dry biomass weight used in analyses. Biomass collected by hand harvest was sorted to species, dried (65°C for 72 hours), and weighed.

### Data analysis

Data were analyzed using SAS 9.2 for Windows. Differences in biomass production between switchgrass and prairie plantings in the GLBRC field surveys were analyzed with t-tests within years because not all plots were repeated for both years. Biomass production estimated from the tractor harvest of the LTER experiment was analyzed with a repeated-measures Analysis of Covariance (ANCOVA) using planted species richness as the covariate and year as the repeated-measure.

We completed several analyses from the LTER experiment hand harvest data. We analyzed the relationship between biomass production and planted species richness with an ANCOVA using seeding density as the covariate, planted species richness as a main plot treatment, and species identity as a nested treatment. Only the 6 grass species planted into both the 6-species and 18-species richness treatments were included in this ANCOVA because only these 6 species were present in both treatments. We calculated species’ expected and observed percent of total biomass based on the initial seeding density or observed biomass production, respectively. We calculated Simpson’s evenness from observed biomass production [28] using the equation [(1 / ∑ p_s_^2^) / S], where p_s_ is the percent of total biomass made up of each species harvested from the plot and S is the number of species harvested from the plot.

The data in the analyses were normally distributed and no transformations were necessary, except for 2012 individual species biomass in the LTER experiment (relationship between seeding density and biomass production), where the data were log transformed. The SAS code for all analyses is included in S3 Table.

## Results

**Fig 1:**
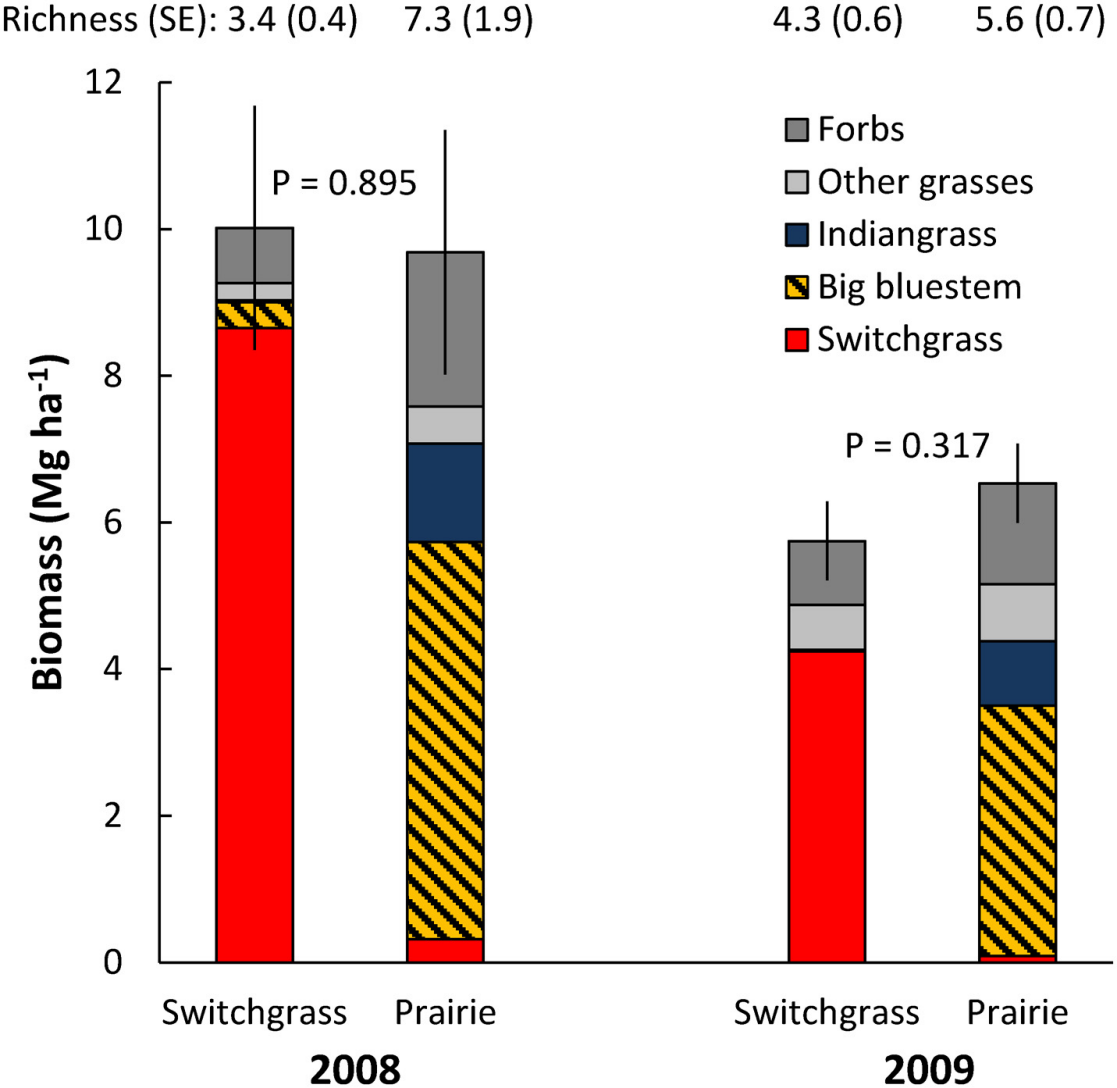
Biomass production from GLBRC field surveys. Sites were planted to switchgrass monocultures or diverse prairie species mixes. Biomass was collected from hand harvests. All error bars are ±1 SE of total biomass; P-values are for within-year comparisons.

**Fig 2:**
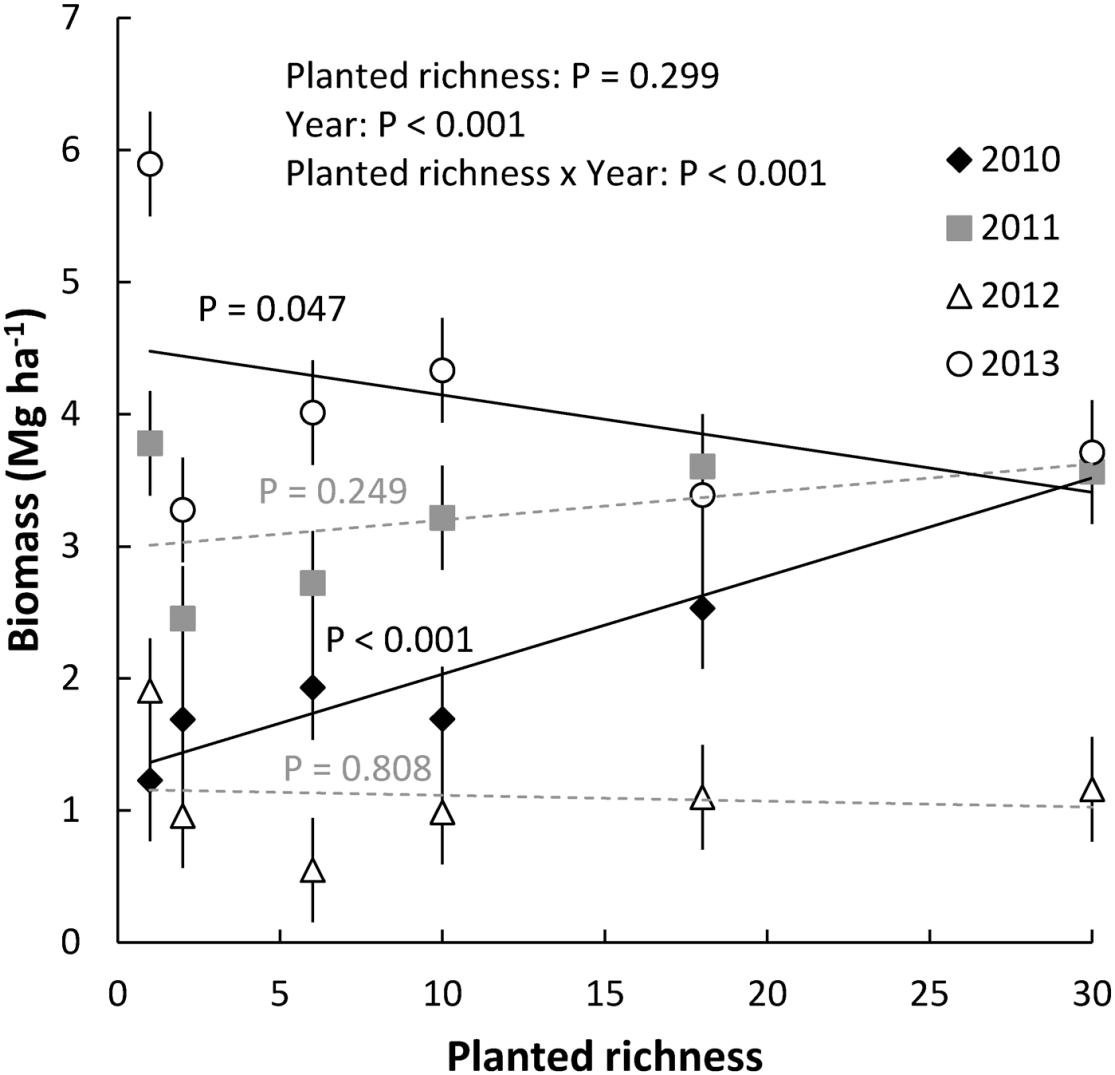
Relationship between the number of planted species and biomass production in the LTER experiment. Biomass was collected from tractor harvests. All error bars are ±1 SE; P-values are for comparisons within years.

In the GLBRC field surveys, we did not find significant differences between biomass production in switchgrass (*Panicum virgatum*) and higher species richness prairie plantings (Fig 1). The composition and relative abundances of species differed between sites planted to switchgrass and a mixture of prairie species, but invasion of non-planted species prevented switchgrass stands from remaining as monocultures. Switchgrass made up between 74% and 86% of biomass in the plots planted to switchgrass, but observed species richness was not much lower in the switchgrass than prairie plantings (Fig 1). For the prairie plots, switchgrass made up less than 4% of the biomass, and most of the remaining biomass was composed of big bluestem (*Andropogon gerardii*) and other native C_4_ grasses (Fig 1). In the LTER experiment there was a significant positive relationship between the number of planted species and biomass production in the first year of sampling (2010; three years after planting), but in subsequent years there was either no significant relationship or a significant negative relationship between species richness and biomass production (Fig 2). Also, there was not a significant relationship between observed species richness and biomass production in the GLBRC field surveys and LTER experiment (S1 Fig).

We found approximately 4-fold more 2012 biomass was collected from the same plots with hand harvest compared to tractor harvest (Fig 2 vs. S1B Fig), even though the relative amounts collected were similarly affected by diversity treatments. Collecting more biomass by hand than with a tractor harvest is similar to other studies [29].

**Fig 3:**
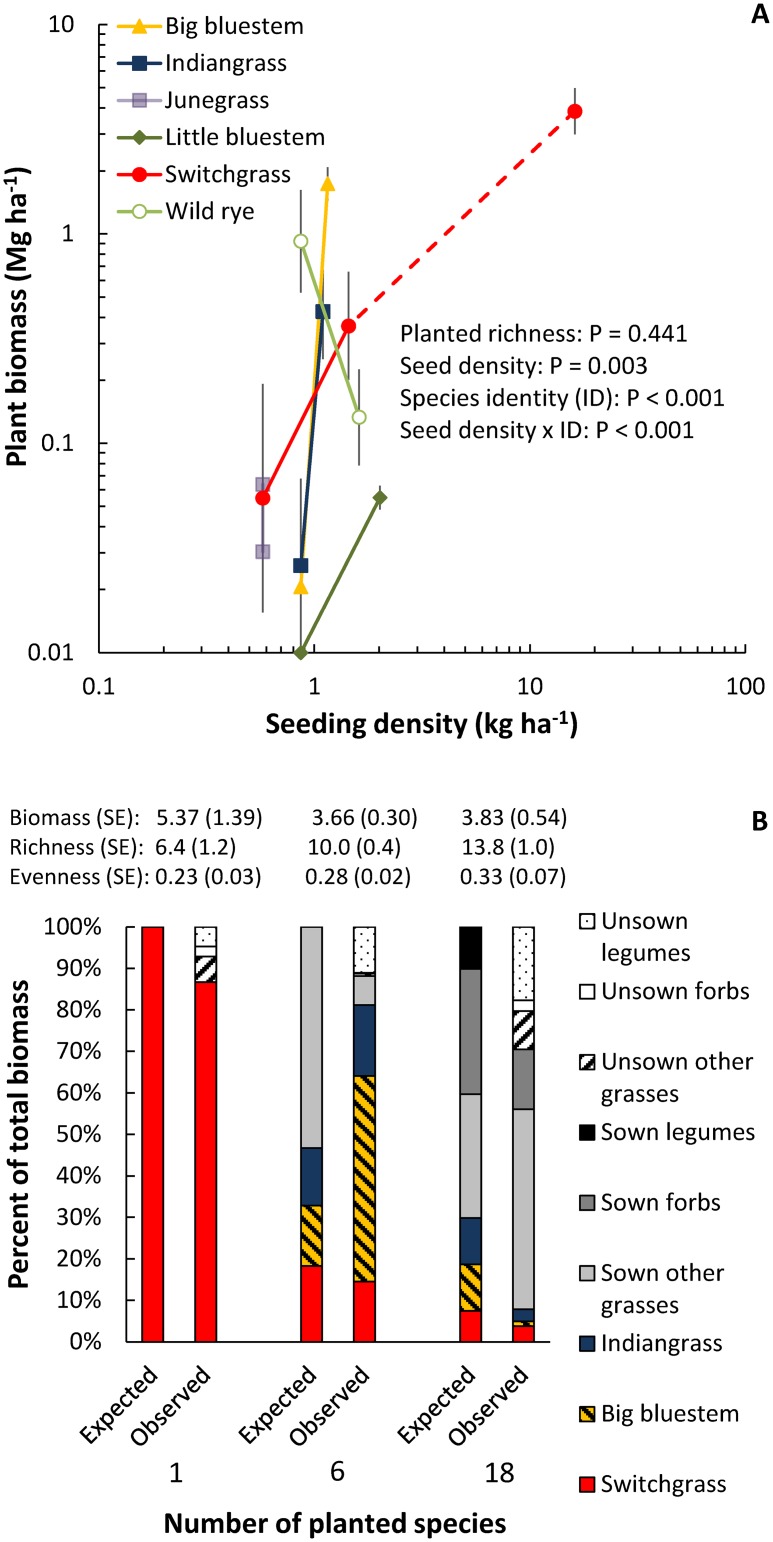
Response of individual species biomass production to seeding density in the LTER experiment. (A) Relationships between seeding density and 2012 biomass production. The points connected by lines show biomass production of species that were planted at different densities in different diversity treatments (only the data points connected by solid lines were included in the analysis, but the switchgrass monoculture is shown to include all the data from each of the species). All error bars are ±1 SE. (B) The expected biomass production of different species planted in the LTER experiment based on the weight of seed added compared to observed biomass production in 2012. Biomass in (A) and (B) was collected from hand harvests.

Because similar total seed mass was used to establish the richness treatments in the LTER experiment, individual species were planted at lower seed densities in the higher species richness treatments (see Table 2). To determine if seeding density was related to biomass production, we compared production of individual species to the seeding density used to establish the different diversity treatments. For the 6 grass species planted into the 6- and 18-species richness treatments, average biomass production was positively correlated with seeding densities, even though this relationship did not hold for all species (significant seed density x species identity interaction; Fig 3A). This suggests that species composition and seeding density of grasses is important in determining total biomass production in diverse plantings of bioenergy crops (Fig 3B).

## Discussion

In our studies, we did not find a persistent positive relationship between species richness and biomass production. In the LTER experiment there was only a positive relationship between planted species richness and biomass production in the first year of sampling (2010). In subsequent years, this relationship was not significant or was negative. In the GLBRC field surveys, there was no significant difference in production between the fields planted to switchgrass (*Panicum virgatum*) monocultures and diverse prairie mixtures. This is one of the first tests of the potential role of biodiversity to produce more biomass in bioenergy plantings using species mixes and seeding densities more realistic to agriculture [see also 18]. The lack of a relationship between plant richness and biomass production in our field surveys and experiment challenges the presumption that results of biodiversity-productivity studies can be directly translated to agriculturally realistic planting designs and management [12,15].

Although we did not find a positive relationship between plant species richness and biomass production, plantings with higher species richness may provide other ecosystem services desired by producers, such as increased stability and better habitat for pollinators, wildlife, and pest suppressing insects [4]. These other services may provide enough incentive for some farmers to manage for high species richness, especially if monetary costs can be minimized by using low densities of expensive seed or by encouraging natural dispersal of species. However, farmers will likely care first and foremost about biomass production, and it is therefore important to determine why increased richness did not increase biomass production in our studies and how future plantings with high species richness might produce more biomass.

### Why did production not increase with plant species richness?

We identified three potential explanations for why we did not observe a positive biodiversity-productivity relationship in our field survey and experimental bioenergy plantings. First, switchgrass was always planted in monocultures in our studies, and mixtures were not a random selection from a larger species pool but instead always included all the species that occurred in less diverse mixtures. This planting design should minimize increases in biomass due to the sampling effect [18]. Past studies have found that more diverse mixtures generally do not outperform the most productive monocultures, such as switchgrass monocultures [19]. Although mixtures may produce more biomass as they age across the first decade of growth [19,23], in our studies we did not find evidence for higher biomass production in more diverse mixtures even five or more years after planting. In fact, the relationship between species richness and biomass production was only positive early and became negative over time in the LTER experiment.

A second potential explanation is that non-planted species (weeds) were not removed from low richness treatments in our GLBRC field surveys and LTER experiment, whereas non-planted species were removed (by hand weeding) to maintain species richness treatments in past biodiversity studies. In our GLBRC field surveys and LTER experiment, non-planted species accounted for an average of 20% and 13%, respectively, of the total biomass in the switchgrass monoculture plantings, and non-planted invasion led to little difference in observed richness between switchgrass monocultures and more diverse plantings (see Figs 1 and 3B). The most common weeds in the LTER experiment switchgrass monocultures were *Elymus repens* and *Poa pratensis*, with 42% and 30% of non-planted grass biomass made up of these non-native species, respectively, and *Trifolium pratense* and *Trifolium hybridum*, with 44% and 22% of non-planted non-grass biomass made up of these non-native legumes, respectively. Non-planted biomass in the GLBRC field surveys was not sorted to species. Other studies have shown that the invasion of non-planted species reduced the observed relationship between richness and productivity [17,30] and reduced differences in species evenness (another measure of diversity that can affect ecosystem function; [31]). This appears to also be the case in our studies. Non-planted species may also outcompete or prevent the establishment of planted species [17] and thereby reduce (or mask) intended differences in species richness. Many of our planted species had minimal establishment, and establishment of these species may have been reduced by competition with non-planted species.

A third potential explanation is that seeding density may play an underappreciated role in experimental studies of the relationship between plant species richness and biomass production. When our LTER experiment was initiated, we did not expect seeding density to affect biomass production, but analyses suggest our comparatively low seeding density may have decreased biomass production relative to other biodiversity experiments (see Fig 3A). For example, Tilman et al. [12] used a total seeding density of 100.0 kg ha^-1^ in a grassland species richness experiment established at the Cedar Creek Ecosystem Science Reserve in Minnesota, USA, whereas our LTER experiment used a total seeding density of 7.2 kg ha^-1^ in the highest richness treatment (Table 2 and S2 Table). Other biodiversity experiments have kept the number of seeds per m^2^ constant and planted an equal number of seeds per species. For example, in the BIODEPTH experiments in Europe, all treatments were planted with a total of 2,000 seeds per m^2^ [32]. In the Jena Experiment in Germany, treatments were established with an average of 1,387 seeds per m^2^ to account for differences in germination [33]. Importantly, the Cedar Creek experiment added seeds by weight whereas the BIODEPTH and Jena experiments added seeds by number of seeds per m^2^. The average weight per 1,000 seeds in the Jena experiment was 2.35 g [33], suggesting that approximately 32.5 kg ha^-1^ of seed was added in this experiment and a somewhat higher density was added in the BIODEPTH experiment.

Seeding densities used in bioenergy plantings likely will be constrained by the cost of seed. Using the same seeding density as the Cedar Creek experiment would cost $37,873 USD per ha in the 18 species mixture of our LTER experiment based on the most inexpensive online retail prices (S2 Table). Wholesale prices might be negotiated for large plantings, but the cost per ha would likely still be quite high. Seeding native North American species at the same density as the BIODEPTH and Jena experiments would cost $25,372 and $17,596 USD retail per ha, respectively (S2 Table). In contrast, the lower seeding density used in our LTER experiment cost $1,766 USD retail per ha in the 30 species treatment and $410 USD retail per ha in the switchgrass monoculture (S2 Table). We recognize that seed prices can be quite variable year to year, but the prices listed above (from October 2013) still provide a good general comparison and are 12% less expensive than seed prices from May 2015 (S2 Table). The cost (and availability) of seed may be an important constraint on both the number of species, and composition of species, used to establish diverse perennial bioenergy plots. Seeding at lower density can reduce costs, but seeding at low density may result in low establishment and biomass production. In our LTER experiment, seed density explained much of the variation in biomass production of individual species (Fig 3A). This can be thought of as a negative sampling effect, whereby seed of one of the most productive species (e.g. switchgrass) is replaced by less productive species at higher richness treatments.

Seeding switchgrass monocultures at densities below 107 pure live seeds m^-2^ (approximately 2.17 kg ha^-1^) can limit production during the first year of establishment [34]. In our LTER experiment 18 species mixtures, switchgrass was seeded at 0.58 kg ha^-1^ (approximately 28.7 seeds m^-2^; conversions between seed weight and number of seeds can be found in S2 Table). This suggests switchgrass production in these treatments was limited by seeding density. Few studies have examined the effects of seeding density on the biomass production of individual species in mixtures (but see [35]), but it seems likely that low seed density will especially affect production when species are competing against other seeded species. Also, seeded species that produce little biomass may compete against species that produce high amounts of biomass and thereby decrease community-level biomass production [36]. Therefore, we hypothesize that seeding high densities of highly productive species is necessary to maximize community-level production.

An acceptable cost of seed obviously depends on the profitability of bioenergy biomass, which is currently difficult to estimate because an active market does not exist. However, shortly before 2010, Michigan bioenergy biomass was estimated to be worth $19–110 USD per Mg with a most likely value around $60 per Mg [37]. This price of biomass was well below the 10-year breakeven price for switchgrass and prairie biomass (compared to corn) given input costs similar to our LTER experiment [37], which means that higher seeding costs would make plantings unprofitable.

### Other considerations

Several other issues that may have reduced the relationship between biodiversity and production in our study are important to consider in future studies. For example, higher diversity plantings will likely include a broader range of plant growth forms, and species with prostrate growth forms will not be harvested by a tractor cutting biomass at 10 cm above the ground. These short statured species may provide important ecosystem services, but it will be important to determine how the height of vegetation affects how much biomass cannot be harvested by tractor [see also 25].

From a more theoretical perspective, most studies which have found a positive relationship between species richness and production have at least partly attributed this relationship to complementarity among species. Complementarity between species could be utilized to create designer species mixtures that might be expected to produce more than switchgrass monocultures. For example, planting grasses in combination with legumes can increase biomass production [12], and planting species that primarily grow at different periods of the growing season (C_4_ vs. C_3_) may also increase biomass production. Future research should seek to create designer planting mixes that maximize complementarity. We cannot directly test the role of complementarity in our study because we have not grown each species in monoculture, but we did not observe that seeding more functional groups than just switchgrass (a C_4_ grass) increased biomass production. However, legumes and non-planted species were present even where only switchgrass was seeded (Figs 1 and 3B), which is a reminder that complete control over species composition is not possible in an agricultural context.

### Can bioenergy plantings be designed to increase the diversity-production relationship?

Bioenergy systems are different from biodiversity-productivity studies in that they are designed to maximize biomass production rather than test a theoretical relationship [38,39]. Therefore, the seeding density and species mixes used will be determined on the basis of potential biomass production, other desired ecosystem services (e.g. pollinator habitat and increased stability), and cost. In our LTER experiment, switchgrass produced large amounts of biomass in monoculture and past studies have identified other prairie species that can produce large amounts of biomass in monoculture [40]. Seeding productive species at high density in diverse plantings could increase biomass production compared to productive monocultures, even though these species may compete strongly and exclude some other species [41,42].

Most current biodiversity plantings are substitutive—they replace part of the seed weight of a species in monoculture with seed of another species. We suggest it is better to use an additive design in bioenergy plantings whereby the seeding density of productive (and inexpensive) seed such as switchgrass and other highly productive species are not reduced in more diverse plantings. This should increase total production. It may be difficult to find seeding densities that allow both high biomass production and high realized plant diversity [41,42], but this should be a focus of future research. Our studies were not long-term enough to examine stability and were not large enough to examine animal diversity, but future research should also examine how varying seeding density affects multiple ecosystem services, such as stability in production and habitat for other species (see also [4]).

## Supporting Information

S1 Fig The relationship between average observed species richness and hand-collected biomass in the GLBRC field surveys and LTER experiment. (PDF)

S1 Table Dataset containing site characteristics and total biomass from the GLBRC field surveys and the LTER experiment. Total biomass from each year of sampling and further information about environmental conditions of sites.
(XLSX)

S2 Table Dataset showing seed density used in our experiment and other experiments. Species used in the Cedar Creek [12] and our LTER experiment, and the seed density planted for each species. We also estimate how much seed would have cost in our LTER experiment if we would have used the same seed densities as the following experiments: [12,32,33].
(XLSX)

S3 Table SAS code and data for all statistical analyses. (DOCX)
