# Supplementary material for: Can the Results of Biodiversity-Ecosystem Productivity Studies Be Translated to Bioenergy Production?
Source: PLoS One. 2015 Sep 11;10(9):e0135253. doi: 10.1371/journal.pone.0135253 (PMC4567130; doi:10.1371/journal.pone.0135253)

Figure S1. The relationship between average observed species richness and hand-collected biomass in the GLBRC field surveys (A) and the 2012 data from the LTER experiment (B). Please note the log scale in (B). P-values refer to the regression between observed species richness and biomass within a particular year.

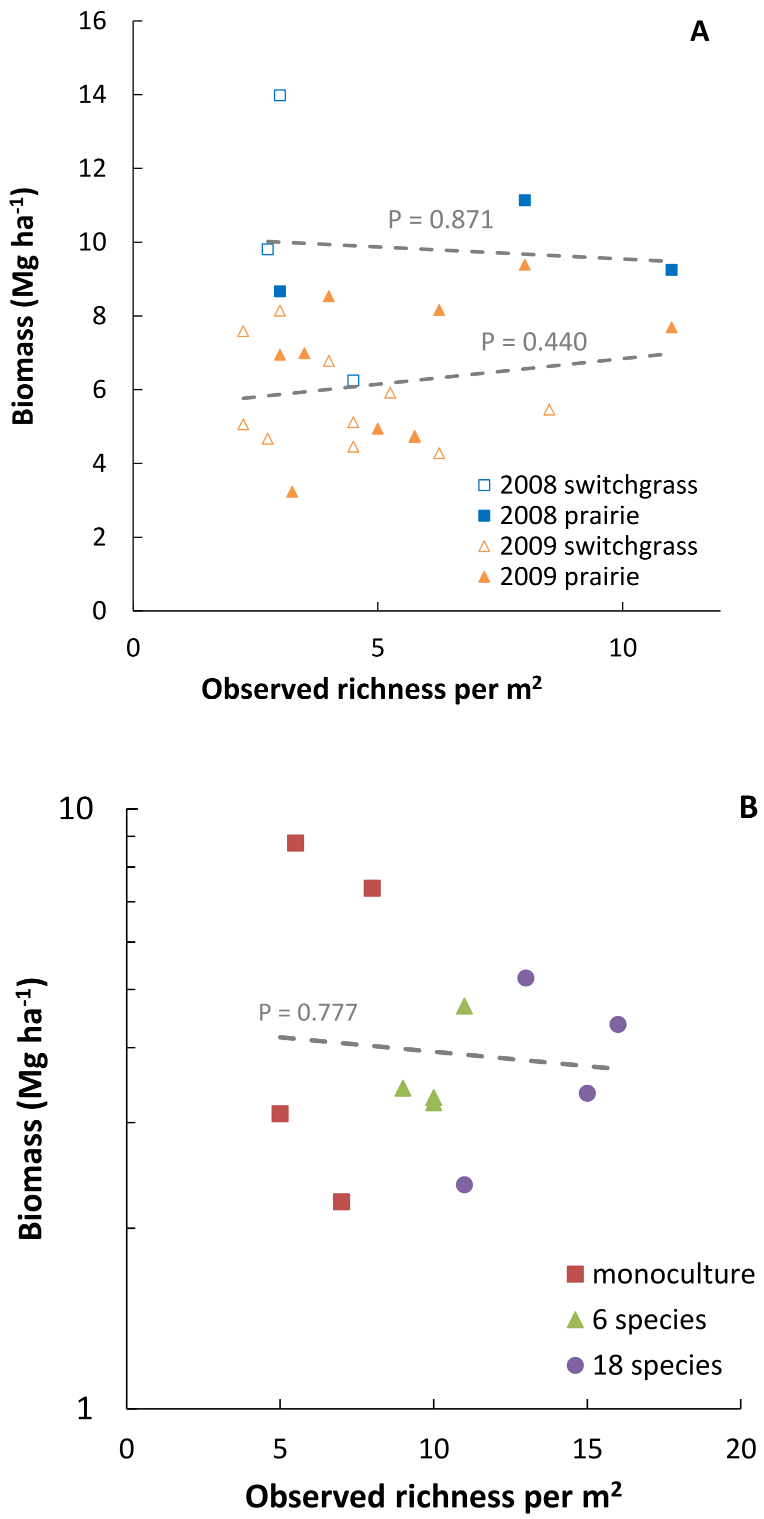

Supplement: S1 Fig — (PDF) [file pone.0135253.s001.pdf]
