# Supplementary material for: Can the Results of Biodiversity-Ecosystem Productivity Studies Be Translated to Bioenergy Production?
Source: PLoS One. 2015 Sep 11;10(9):e0135253. doi: 10.1371/journal.pone.0135253 (PMC4567130; doi:10.1371/journal.pone.0135253)
Supplement: S3 Table — (DOCX) [file pone.0135253.s004.docx]

*Analyses for Dickson and Gross “Can the results of biodiversity-ecosystem productivity studies be translated to bioenergy production?”

*analysis for Figure 1;

**data** GLBRC_field_surveys;

input Year Code$ Treatment Site Richness_m2 Biomass_Mg_ha;

cards;

2008 SW02 1 2 4.5 6.250375

2008 SW07 1 7 2.75 9.80935

2008 SW08 1 8 3 13.985675

2008 PR03 2 3 8 11.135025

2008 PR06 2 6 11 9.247325

2008 PR10 2 10 3 8.666275

2009 SW01 1 1 6.25 4.276625

2009 SW02 1 2 4.5 5.11715

2009 SW07 1 7 2.75 4.672975

2009 SW08 1 8 3 8.1471

2009 SW09 1 9 4.5 4.453825

2009 SW10 1 10 2.25 7.5852

2009 SW11 1 11 8.5 5.459725

2009 SW12 1 12 5.25 5.917275

2009 SW13 1 13 2.25 5.058775

2009 SW14 1 14 4 6.77905

2009 PR02 2 2 3.25 3.23415

2009 PR03 2 3 8 9.38715

2009 PR04 2 4 5.75 4.74585

2009 PR06 2 6 11 7.69085

2009 PR07 2 7 4 8.5347

2009 PR08 2 8 3.5 6.98935

2009 PR09 2 9 5 4.94345

2009 PR10 2 10 3 6.945725

2009 PR11 2 11 6.25 8.16405

2009 PR12 2 12 5.75 4.710375

;

*The code immediately below is for the analyses in Figure 1;

**proc** **mixed**;

class Year Code Treatment Site;

*Input year = 2008 or year = 2009 to analyze the year of interest;

where Year = **2008**;

model Biomass_Mg_ha = Treatment

/ddfm=kr outp = GLBRC residual;

**run**;

*The code immediately below is for the analyses in Figure S1A;

**proc** **mixed**;

class Year Code Treatment Site;

*Input year = 2008 or year = 2009 to analyze the year of interest;

where Year = **2008**;

model Biomass_Mg_ha = Richness_m2

/ddfm=kr outp = GLBRC residual;

**run**;

*analysis for Figure 2;

**data** LTER_experiment_tractor_harvest;

input year plot block number_species Biomass_Mg_ha;

cards;

2010 107 1 1 1.0162

2010 208 2 1 1.46133

2010 309 3 1 1.04214

2010 407 4 1 .

2010 103 1 2 1.56981

2010 211 2 2 2.16135

2010 307 3 2 1.06822

2010 408 4 2 1.9566

2010 102 1 6 2.47414

2010 201 2 6 1.65629

2010 308 3 6 0.726602

2010 409 4 6 2.86451

2010 104 1 10 2.40747

2010 206 2 10 0.574649

2010 306 3 10 1.61599

2010 410 4 10 2.17208

2010 101 1 18 .

2010 209 2 18 2.12738

2010 311 3 18 1.72893

2010 411 4 18 3.23227

2010 109 1 30 2.73272

2010 212 2 30 4.9235

2010 305 3 30 3.24654

2010 412 4 30 3.63572

2011 107 1 1 3.30934

2011 208 2 1 4.8564

2011 309 3 1 2.91413

2011 407 4 1 4.04547

2011 103 1 2 2.56965

2011 211 2 2 2.70955

2011 307 3 2 1.1288

2011 408 4 2 3.40008

2011 102 1 6 3.24333

2011 201 2 6 2.80322

2011 308 3 6 1.53345

2011 409 4 6 3.30636

2011 104 1 10 4.00247

2011 206 2 10 1.60273

2011 306 3 10 3.84833

2011 410 4 10 3.41137

2011 101 1 18 3.41275

2011 209 2 18 3.93035

2011 311 3 18 3.18424

2011 411 4 18 3.89356

2011 109 1 30 3.57019

2011 212 2 30 3.81184

2011 305 3 30 3.18751

2011 412 4 30 3.6967

2012 107 1 1 2.42346

2012 208 2 1 1.83596

2012 309 3 1 1.22842

2012 407 4 1 2.13747

2012 103 1 2 2.02292

2012 211 2 2 0.567902

2012 307 3 2 0.308303

2012 408 4 2 0.933095

2012 102 1 6 0.965585

2012 201 2 6 0.372373

2012 308 3 6 0.0972703

2012 409 4 6 0.758154

2012 104 1 10 1.64024

2012 206 2 10 0.402862

2012 306 3 10 0.440853

2012 410 4 10 1.4644

2012 101 1 18 1.01837

2012 209 2 18 1.45568

2012 311 3 18 0.996831

2012 411 4 18 0.930368

2012 109 1 30 1.17255

2012 212 2 30 0.786786

2012 305 3 30 1.10147

2012 412 4 30 1.58658

2013 107 1 1 6.45339

2013 208 2 1 6.03024

2013 309 3 1 4.55751

2013 407 4 1 6.54386

2013 103 1 2 4.18312

2013 211 2 2 4.02547

2013 307 3 2 2.67899

2013 408 4 2 2.21942

2013 102 1 6 5.40908

2013 201 2 6 5.28208

2013 308 3 6 3.50311

2013 409 4 6 1.85334

2013 104 1 10 5.15792

2013 206 2 10 3.52459

2013 306 3 10 4.90182

2013 410 4 10 3.73914

2013 101 1 18 5.81292

2013 209 2 18 3.35248

2013 311 3 18 2.27835

2013 411 4 18 2.10867

2013 109 1 30 3.63724

2013 212 2 30 3.11411

2013 305 3 30 4.34657

2013 412 4 30 3.74457

;

**proc** **mixed**;

class year plot block;

model Biomass_Mg_ha = Block number_species Year Year*number_species

/ddfm=kr outp = LTER residual;

random Block;

repeated / type=cs subject=plot;

estimate 'number_species:year 1' number_species **1** year*number_species **1** **0** **0** **0**;

estimate 'number_species:year 2' number_species **1** year*number_species **0** **1** **0** **0**;

estimate 'number_species:year 3' number_species **1** year*number_species **0** **0** **1** **0**;

estimate 'number_species:year 4' number_species **1** year*number_species **0** **0** **0** **1**;

**run**;

*analysis for Figure 3A;

**data** LTER_2012_hand_harvest;

input block richness block_richness$ species$ species_identity Biomass_Mg_ha seed_kg_ha;

log_Biomass_Mg_ha = log10(Biomass_Mg_ha+**0.01**);

log_seed_kg_ha = log10(seed_kg_ha);

cards;

1 6 1_6 Big_bluestem 1 1.5913 1.15

2 6 2_6 Big_bluestem 1 2.7137 1.15

3 6 3_6 Big_bluestem 1 1.1097 1.15

4 6 4_6 Big_bluestem 1 1.8426 1.15

1 18 1_18 Big_bluestem 1 0.1709 0.87

2 18 2_18 Big_bluestem 1 0 0.87

3 18 3_18 Big_bluestem 1 0 0.87

4 18 4_18 Big_bluestem 1 0 0.87

1 6 1_6 Indiangrass 2 0.4023 1.1

2 6 2_6 Indiangrass 2 0.3912 1.1

3 6 3_6 Indiangrass 2 1.5874 1.1

4 6 4_6 Indiangrass 2 0.1145 1.1

1 18 1_18 Indiangrass 2 0.4501 0.87

2 18 2_18 Indiangrass 2 0 0.87

3 18 3_18 Indiangrass 2 0 0.87

4 18 4_18 Indiangrass 2 0 0.87

1 6 1_6 Junegrass 3 0.0484 0.58

2 6 2_6 Junegrass 3 0 0.58

3 6 3_6 Junegrass 3 0 0.58

4 6 4_6 Junegrass 3 0.1367 0.58

1 18 1_18 Junegrass 3 0.1928 0.58

2 18 2_18 Junegrass 3 0 0.58

3 18 3_18 Junegrass 3 0 0.58

4 18 4_18 Junegrass 3 0.7964 0.58

1 6 1_6 Little_bluestem 4 0.0574 2.02

2 6 2_6 Little_bluestem 4 0.0341 2.02

3 6 3_6 Little_bluestem 4 0.0605 2.02

4 6 4_6 Little_bluestem 4 0.0338 2.02

1 18 1_18 Little_bluestem 4 0 0.87

2 18 2_18 Little_bluestem 4 0 0.87

3 18 3_18 Little_bluestem 4 0 0.87

4 18 4_18 Little_bluestem 4 0 0.87

1 6 1_6 Switchgrass 5 0.5019 1.44

2 6 2_6 Switchgrass 5 1.1598 1.44

3 6 3_6 Switchgrass 5 0.4112 1.44

4 6 4_6 Switchgrass 5 0.0591 1.44

1 18 1_18 Switchgrass 5 0.3279 0.58

2 18 2_18 Switchgrass 5 0 0.58

3 18 3_18 Switchgrass 5 0 0.58

4 18 4_18 Switchgrass 5 0.2564 0.58

1 6 1_6 Wild_rye 6 0.0173 1.62

2 6 2_6 Wild_rye 6 0.2055 1.62

3 6 3_6 Wild_rye 6 0.2371 1.62

4 6 4_6 Wild_rye 6 0.2061 1.62

1 18 1_18 Wild_rye 6 0.5552 0.87

2 18 2_18 Wild_rye 6 4.7643 0.87

3 18 3_18 Wild_rye 6 0.7018 0.87

4 18 4_18 Wild_rye 6 0.3664 0.87

;

**proc** **mixed**;

class block richness block_richness species species_identity;

model log_Biomass_Mg_ha = richness log_seed_kg_ha species_identity log_seed_kg_ha*species_identity

/ddfm=kr outp = LTER_2012_individual residual;

repeated / type=cs subject=block_richness;

**run**;

*analysis for Figure S1B;

**data** LTER_2012_hand_harvest_richness;

input Plot Block Biomass_Mg_ha Observed_richness_m2 Planted_richness;

log_Biomass_Mg_ha = log10(Biomass_Mg_ha);

cards;

107 1 2.2119 7 1

208 2 3.1018 5 1

309 3 7.3797 8 1

407 4 8.772 5.5 1

102 1 3.2369 10 6

201 2 4.6915 11 6

308 3 3.4214 9 6

409 4 3.3022 10 6

101 1 2.3623 11 18

209 2 5.2249 13 18

311 3 4.3726 16 18

411 4 3.3563 15 18

;

**proc** **mixed**;

class Block Planted_richness;

model log_Biomass_Mg_ha = Observed_richness_m2

/ddfm=kr outp = LTER_2012_community residual;

**run**;
